# Implementing the TARGET Model in Physical Education: Effects on Perceived Psychobiosocial and Motivational States in Girls

**DOI:** 10.3389/fpsyg.2017.01517

**Published:** 2017-09-05

**Authors:** Laura Bortoli, Maurizio Bertollo, Edson Filho, Selenia di Fronso, Claudio Robazza

**Affiliations:** ^1^Behavioral Imaging and Neural Dynamics Center, Department of Medicine and Aging Sciences, “G. d’Annunzio" University of Chieti-Pescara Chieti-Pescara, Italy; ^2^School of Psychology, University of Central Lancashire Preston, United Kingdom

**Keywords:** achievement goal theory, self-determination theory, physical education, motivation, emotion, psychobiosocial states

## Abstract

Grounded in achievement goal and self-determination theories, the purpose of this study was to investigate the effects of mastery and performance climate interventions on students’ psychobiosocial (PBS) states and self-determined motivation. A first study was conducted to determine the validity of the measures. In a second study, two groups of female students (*N* = 65, 14–15 years of age) took part in the investigation. A mastery-performance group participated in eight task-involving lessons and then in another set of eight ego-involving lessons. A performance-mastery group participated in ego-involving lessons and then in task-involving lessons. Findings revealed that the program was effective in changing PBS states and self-determined motivation in the performance-mastery group. In particular, participants in this group reported lower scores on pleasant/functional PBS states and self-determined motivation after the first phase of the intervention. Furthermore, lower levels of self-determined motivation were maintained after the second phase of the intervention, thereby suggesting detrimental carryover effects.

## Introduction

In a seminal article, Sallis and McKenzie (1991) outlined the role of physical education in the achievement of public health goals, particularly in providing youth with opportunities to develop habitual physical activity behaviors. Since then, increased attention has been given to promoting physical activity in schools, as well as in preparing physical education teachers within a public health perspective (for reviews, see Sallis et al., 2012; McEvoy et al., 2015; Webster et al., 2015). As Biddle et al. (2014) noted, preadolescents are the most active segment of society. Yet, there remains concern that many in this age group have physical activity levels lower than those recommended for good health.

Despite the widely acknowledged health benefits deriving from an active lifestyle, recent studies have shown that physical activity declines during adolescence. This decline is greater and begins earlier in girls than boys across ages 12–15 years (e.g., Nader et al., 2008; Dumith et al., 2011). Based on this evidence, Biddle et al. (2014) conducted a meta-analysis on the effectiveness of interventions to increase physical activity among girls. They found a higher effect size for interventions that targeted just girls rather than boys and girls together, likely due to self-presentation issues such as body image and physical perception (Labbrozzi et al., 2013). Biddle et al.’s (2014) findings suggest that boys and girls may have different needs, with respect to the provision of structured physical activity contexts. Furthermore, Biddle et al.’s (2014) study showed that shorter interventions (i.e., less than 12 weeks) were more effective, probably because of motivational issues arising with longer interventions. To strengthen the evidence base for interventions among young girls, the authors concluded their meta-analysis by recommending future studies with rigorous designs. Similarly, previous research has echoed the need to study the unique psychological dynamics of girls in physical education, sport, and exercise settings (Filho et al., 2014).

In this context, the main purpose of the current study was to examine the effects of two different physical education interventions in changing the perceived motivational climate determined by the teacher and, as a consequence, emotional states and motivation toward physical activity among young girls. The study was grounded in two prominent theories used to understand motivation in physical activity settings, namely, achievement goal and self-determination theories. We also used Hanin’s (2000, 2007) individual zones of optimal functioning (IZOF) model as a theoretical perspective in the assessment of a range of emotional states related to the physical education experience. Finally, we adopted the TARGET model indications (Epstein, 1989) to manipulate the motivational climate. These theoretical approaches have been chosen for the current study owing to their unique contribution to the understanding of the motivation and emotion interplay, and because of the practical relevance of indications stemming from them. So far, no previous research has been conducted using achievement goal, self-determination, IZOF, and TARGET frameworks in a single study. These approaches are briefly reviewed next.

### Achievement Goal Theory

Achievement goal theory is a motivational viewpoint extensively applied in school, exercise, and sport settings (Nicholls, 1984, 1992; Ames, 1992; Treasure, 2001; Roberts and Treasure, 2012). According to this framework, the teacher can establish two types of motivational climate: (a) the mastery (task-involving) climate, wherein personal improvement, effort, and learning prevail; and (b) the performance (ego-involving) climate, in which comparison and rivalry among pupils are encouraged (Ames, 1992). Several studies have been conducted using this theoretical framework. Most of them were cross-sectional, and directed at identifying the correlates of perceived mastery or performance climates. Findings generally provide support for positive links among a mastery climate, enjoyment (Liukkonen et al., 2010), and adaptive motivational processes (Barkoukis and Hagger, 2013), whereas a performance climate relates to negative or maladaptive consequences (see Duda et al., 2014). For instance, perception of a mastery climate was found to be related to perception of lesson usefulness and greater interest (Papaioannou, 1994), positive attitude toward the class, high perceived ability, and feelings of satisfaction (Treasure, 1997; Treasure and Roberts, 2001). Motivational climate was also found to be more influential than individual goals in stimulating pupil’s interest in physical education (Cury and Biddle, 1996). Moreover, research has shown the positive impact of a mastery climate on intention to be physically active, practice sport, and develop self-determined motivation to engage in out-of-school physical activity (Theodosiou and Papaioannou, 2006; Sproule et al., 2007; Barkoukis and Hagger, 2013; Jaakkola et al., 2017).

### Self-determination Theory

According to self-determination theory (Deci and Ryan, 1985, 2000; Ryan and Deci, 2017), various types of motivation, ranging from high to low levels of self-determination, underlie human behavior: intrinsic motivation (IM), integrated regulation, identified regulation (IR), introjected regulation, external regulation (ER), and amotivation (AM). In the current study we used the Situational Motivation Scale (SIMS; Guay et al., 2000) to assess IM, IR, ER, and AM. Intrinsically motivated individuals take part in an activity in order to experience pleasure and satisfaction derived from the participation itself. Identified and external regulations are both forms of extrinsic motivation. The former is observed when a behavior is performed in order to obtain benefits deemed important and worthy; the latter is manifested when participation is regulated by reward, or is intended to avoid negative consequences. Finally, AM refers to a relative lack of both intrinsic and extrinsic forms of motivation, such as when individuals feel that being involved in an activity is worthless. Research findings in physical education showed that an autonomy-supportive context promotes autonomous motivation, participation, enjoyment, vitality, effort, and persistence, as well as healthy lifestyles and physical activity inside and outside of classes (Taylor et al., 2010; Mouratidis et al., 2011; Standage et al., 2012; for a review, see Ryan and Deci, 2017).

Several studies on motivation in physical education have considered achievement goal theory together with self-determination theory. For instance, Jaakkola and Liukkonen (2006) found a task-involving intervention to positively influence students’ self-determined motivation. Jaakkola et al. (2017) also showed that students who perceived a higher mastery-oriented climate exhibit greater forms of autonomous motivational regulations compared to those who perceived a lower mastery-oriented climate. They also enjoyed physical education activities more and had lower AM. In contrast, those students who perceived a highly performance-oriented climate were more externally motivated and amotivated. Sproule et al. (2007) found perceptions of a mastery climate in physical education to foster both IM and intentions to be physically active in 14–16 years old students.

Drawing from both achievement goal theory and self-determination theory, Duda et al. (2014) defined the motivational climate in physical education as the social psychological environment created by teachers in the manner they provide feedback, evaluate, and organize lessons. In this integrative approach, Duda (2013) conceptualizes motivational climate as being more *empowering* when the social environment is highly task-involving, autonomy supportive, and socially supportive (i.e., when the teacher demonstrates care and respect for students regardless of their level of engagement or performance). Conversely, a more *disempowering* motivational climate occurs when teachers’ behaviors are highly ego-involving and controlling. In fact, previous research in physical education supports the notion that pupils’ interest, enjoyment, and vitality depend on the motivational climate (Mouratidis et al., 2011). Findings showed that pupils enjoyed classes more and felt more energized when their teachers adopted a need-supportive teaching style in which the basic psychological needs for autonomy, competence, and relatedness were satisfied. To optimally motivate students for physical education, it is therefore critical to satisfy their psychological needs for autonomy, competence, and relatedness. Teachers should be autonomy-supportive, structure the environment appropriately, create a warm relationship with their students, and foster positive emotional states (for a review on self-determination in physical education, see Van den Berghe et al., 2014).

### Psychobiosocial States

Emotions in achievement settings have attracted increasing attention over the years because they are critically important for students’ motivation, learning, performance, and well-being (Pekrun and Linnenbrink-Garcia, 2014). Pleasant emotions are contended to positively influence self-regulatory motivational and cognitive processes, such as creative, flexible, and holistic ways of solving problems, which are relevant to academic achievement and personal growth (Pekrun et al., 2009). In contrast, unpleasant emotions would engender more analytical, detailed, and inflexible ways of elaborating on information. Among the theoretical models that have been used to study emotions in achievement settings, Pekrun’s (2006) control-value theory is an integrative approach in the analysis of a range of emotions experienced in several contexts, such as academic settings, sport, and professions. In this theory, emotions are viewed as sets of interrelated psychological processes, whereby emotional, cognitive, motivational, and physiological components are fundamental. Anxiety, for example, can entail emotional (feeling distressed), cognitive (worry), motivational (withdrawal tendencies), and physiological (peripheral activation) components (Pekrun et al., 2011). A large body of literature supports the predictions from this theory (for a meta-analysis, see Huang, 2011). A consistent finding is that mastery goals (i.e., attaining mastery standards and developing competence) relate positively to students’ enjoyment and negatively to students’ boredom and anger. In contrast, performance-avoidance goals (i.e., not performing poorly relative to others) are positively related to anxiety and shame (Goetz et al., 2016).

In the sport setting, a model that shares some features with Pekrun’s (2006) control-value theory is Hanin’s (2000, 2007) IZOF model, which is one of the most widely applied theoretical frameworks to the study of emotional experiences related to athletic performance. The model has also been applied to the physical education context (for review, see Ruiz et al., 2017). In both theoretical perspectives, emphasis is placed on the emotional, cognitive, motivational, and physiological components underlying individual experiences. The IZOF model, in particular, is a holistic approach to understanding individuals’ experiences. The IZOF model incorporates a large array of idiosyncratic performance-related states, named psychobiosocial (PBS) states, which include at least seven emotional and non-emotional interactive components. These components are: (a) emotional, cognitive, and motivational (psychological states); (b) bodily (physiological) and motor-behavioral (biological states); and (c) operational and communicative (social states). It is worth mentioning that volition has been recently included as a component of PBS states (Hanin, 2010). Consistent with the IZOF model, people can perceive these states as pleasant or unpleasant, and as functional (i.e., facilitative to performance) or dysfunctional (i.e., debilitative to performance; e.g., Bortoli et al., 2009, 2011; Robazza et al., 2016; Ruiz et al., 2016). In particular, the emotional component can be individually perceived as pleasant or unpleasant and exerting functional or dysfunctional effects toward performance, whereas all other components can be perceived as functional or dysfunctional.

Students’ PBS states have been evaluated in a number of studies in physical education (e.g., Robazza et al., 2006; Bortoli and Robazza, 2007). PBS states have been shown to mediate the relationship between motivational climate and individuals’ motivation as conceived within the self-determination theory (Bortoli et al., 2014). In particular, a perceived mastery atmosphere was linked to youngsters’ IM and IR (two forms of motivation reflecting high levels of self-determination) through the mediation of pleasant/functional PBS states. On the other hand, a perceived performance climate was related to ER and AM (reflecting low levels of self-determination) through the mediation of unpleasant/dysfunctional PBS states.

### The TARGET Model

The TARGET model, which draws support from both the achievement goal and self-determination theories, has been proposed as an effective approach to create adaptive motivational climates (Epstein, 1989). Given the empirical evidence on the positive effects of a mastery climate, physical educators are suggested to adopt instructional strategies to improve a mastery atmosphere in classroom settings. The acronym TARGET refers to the six different dimensions of the model: *Tasks*, *Authority*, *Recognition*, *Grouping*, *Evaluation*, and *Time*. Each dimension involves strategies intended to foster task engagement and reduce social comparison. In a mastery climate pupils work on different tasks, are allowed to work at their own ability level, and are encouraged to participate in decisions regarding various aspects of the lesson. Moreover, they have opportunities to receive reward based on individual progress and work in mixed-ability small flexible groups. Pupils are evaluated based on self-referenced criteria (personal goals achievement, participation, and effort), and have flexible timeline to complete a given task, according to their specific needs and skills. This is in line with both the achievement goal and self-determination theories, which highlight the importance of focusing on mastering tasks and personal improvement rather than on striving to outperform others. An autonomy-supportive environment (i.e., involving pupils in making decisions about their learning and developing their self-management skills) meets individual’s psychological needs and promotes higher levels of self-regulation, thereby fostering IM and a prolonged engagement in physical activity.

The TARGET model has been applied in physical education. In a meta-analysis, Braithwaite et al. (2011) summarized findings from 22 research papers describing TARGET model interventions. Collectively, results showed adaptive outcomes for groups experiencing a mastery climate, and negative outcomes for performance climate conditions. In this meta-analysis there were only three studies involving 14–15 years old students, thus illustrating the need to conduct further research with this age cohort. In a recent work with teenagers, Bortoli et al. (2015) examined the effects of a climate manipulation intervention on emotional consequences in 14–15 years old physical education female students. Lessons were grounded in the TARGET model to create a mastery or performance climate. After the intervention, lower scores in pleasant/functional PBS states and higher scores in unpleasant/dysfunctional PBS states were observed in the participants in the performance group.

### Study Purposes

In light of previous findings regarding motivational climate interventions, the aim of our study was to examine the effects of different climate (mastery and performance) on both PBS states and self-determined motivation. We also sought to determine the effects of a particular motivational climate change when an opposite motivational climate is adopted. To these purposes, a group of pupils was involved in a mastery climate and later in a performance climate, whereas another group was initially involved in a performance climate and then in a mastery climate. We formulated the following hypotheses:

Hypothesis 1: Climate manipulations would determine corresponding changes in students’ perceptions of the motivational climate (Bortoli et al., 2015). Specifically, a mastery climate was expected to enhance the students’ perceptions of a mastery atmosphere and decrease perceptions of a performance atmosphere. Likewise, a performance climate was predicted to enhance students’ perceptions of a performance atmosphere and decrease perceptions of a mastery atmosphere.
Hypothesis 2: A mastery climate would increase the levels of pleasant/functional PBS states and self-determination, whereas a performance climate would increase the levels of unpleasant/dysfunctional PBS states and decrease self-determination.
Hypothesis 3: Switching to an opposite motivational climate would lead to changes on PBS states and self-determined motivation. This contention was based on the findings by Ward et al. (2008) who examined the effects of increased autonomy on self-determination and physical activity levels of adolescent girls through a counterbalanced quasi-experimental study similar to the present study. Ward et al. (2008) found that the girls who experienced a choice unit first (a condition fitting a mastery climate) and then were denied the opportunity to make choices (as it occurs in a performance climate) reported lower levels of self-determination at the end of the intervention, probably due to dissatisfaction deriving from a more teacher-controlled environment. Conversely, a no-choice unit followed by a choice unit increased self-determination in a second group of girls.

To the best of our knowledge, counterbalanced experimental designs have not been applied so far to investigate the relationships among motivational climate, PBS states, and self-determined motivation in physical education. Before carrying out the experimental study, we conducted an investigation (Study 1) to assess the factorial validity of the measures. In Study 1, we were also interested in investigating whether PBS states mediate the link between students’ mastery and performance climate perception and motivation (Bortoli et al., 2014). Study 2 was specifically planned to investigate the main objectives and hypotheses stated above.

## Study 1

### Method

#### Participants

Using Soper’s (2017) software, *a priori* sample size calculation for structural equation modeling, anticipating a medium effect size of 0.30, a desired power level of 0.80 and *p* < 0.05, for 2 latent variables and 14 observed variables (as related to the most complex model in this study), suggested a minimum sample size of 90. Our sample consisted of 184 female students aged 14–15 years (*M* = 14.60, *SD* = 0.49) drawn from four high schools in northeastern Italy. All the schools were located in middle socioeconomic and cultural areas. Students participated in physical education lessons as a required course twice a week, for 50 min each lesson, during their first year of high school. Permission for data collection was obtained from the headmasters, and then from the students and their parents or guardians who signed an informed consent in accordance with the Declaration of Helsinki. Ethical approval for the study was obtained from the university’s ethics committee with anonymity and confidentiality being assured for all the participants.

#### Measures

Assessment included the individual’s perceptions of the motivational climate, PBS states, and motivation.

##### Perceived motivational climate

We administered the Teacher-Initiated Motivational Climate in Physical Education Questionnaire (TIMCPEQ; Papaioannou, 1998) to assess the individual’s perceptions of the motivational climate. The TIMCPEQ consists of two 6-item scales. The teacher-initiated mastery orientation scale measures the teacher’s emphasis on skill mastery and effort (e.g., “The physical education teacher is most satisfied when every student learns something new”), while the teacher-initiated performance orientation scale measures the teacher’s emphasis on social comparison and competition (e.g., “Only the students with the best records are rewarded”). With the stem “In this physical education class,” pupils are asked to indicate their responses on a 5-point scale ranging from 1 = *strongly disagree* to 5 = *strongly agree*. The TIMCPEQ, translated and adapted in the Italian language (Bortoli et al., 2008), was administered to boys and girls aged from 11 to 14 years. Confirmatory factor analysis gave support to the two-dimensional structure of the questionnaire. Cronbach α values of the mastery and performance scale scores were 0.79 and 0.70, respectively.

##### Psychobiosocial states

A 14-item list of pleasant/functional descriptors (seven items) and unpleasant/dysfunctional descriptors (seven items) was used to gauge the students’ PBS states (Bortoli and Robazza, 2007). The descriptors derived from an existing lists of adjectives that has been adopted to assess emotional experiences in youth sport and physical education (e.g., Robazza and Bortoli, 2005; Robazza et al., 2006). Noteworthy, this list of adjectives is based on seven PBS components conceptualized within the IZOF model (Hanin, 2000; see Robazza et al., 2016; Ruiz et al., 2016). An item (discrete PBS state) includes two or three descriptors, rather than just one descriptor, in order to convey a clear and direct representation of an emotional experience occurring in the physical education domain. Pleasant/functional or unpleasant/dysfunctional items for each PBS component are: “happy, joyful, cheerful,” and “depressed, sad” (emotion); “convinced, resolute, purposeful,” and “inactive, sluggish, passive” (cognition); “involved, determined, committed,” and “unmotivated, disengaged” (motivation); “physically fresh, reactive,” and “tense, stiff muscles” (bodily reaction); “active, dynamic,” and “awkward, clumsy” (movement); “capable, proficient, effective,” and “doubtful, unsure, uncertain” (performance); “socializing, collaborative,” and “lonely, isolated” (communication). Participants are asked to rate each item on a five-point scale ranging from 0 = *not at all* to 4 = *very, very much*, thinking of how they currently feel within their physical education context. Bortoli and Robazza (2007) found a two-factor solution (i.e., pleasant and unpleasant dimensions) to be acceptable. Cronbach α values were 0.84 for the pleasant scale scores and 0.72 for the unpleasant scale scores.

##### Motivation

The Situational Motivation Scale (SIMS; Guay et al., 2000), grounded in the self-determination theory (Deci and Ryan, 1985), was proposed to assess the constructs of IM, IR, ER, and AM. The SIMS is a 16-item scale composed of four factors with four items each, according to the theorized constructs. The stem of items is “…please circle the number that best describes the reason why you are currently engaged in this activity.” Each item is rated on a 7-point scale ranging from 1 = *does not correspond at all* to 7 = *corresponds exactly*. Cronbach’s α values for the subscales were: intrinsic motivation = 0.95 (e.g., “I think that this activity is pleasant”), identified regulation = 0.80 (e.g., “I believe that this activity is important for me”), external regulation = 0.86 (e.g., “it is something that I have to do”), and amotivation = 0.77 (e.g., “I do this activity but I am not sure if it is worth it”). The SIMS was translated from English to Italian and backward by five researchers and a native English speaker professional.

#### Procedure

Two months after the start of the academic year, assessment was carried out in groups of four or five pupils in a secluded classroom near to the physical education facilities, without the presence of the teacher. After having assured participants about confidentiality of individual results, they were asked to complete the questionnaires thinking about their current experience in physical education. Anti-social desirability instructions were presented placing emphasis on the importance of being honest while responding to the surveys.

### Data Analysis

Data were screened for missing cases, skewness, kurtosis, and multivariate outliers (Tabachnick and Fidell, 2013). Descriptive statistics, Pearson product-moment correlation coefficients, reliability Cronbach’s alpha values, and composite reliability values of the latent variables were computed. The factorial validity of the measures was examined through Confirmatory Factor Analysis (CFA). CFA models were estimated using the maximum likelihood parameter estimates (MLM) with standard errors and a mean-adjusted chi-square test statistic that is robust to non-normality (Muthén and Muthén, 2012). The MLM estimator is most appropriately used with continuous and non-normally distributed data (Byrne, 2012). All data analyses were conducted in Mplus version 7.31 (Muthén and Muthén, 2012). Following the suggestions of several researchers (Hu and Bentler, 1999; MacCallum and Austin, 2000), different fit indices were chosen to assess model fit: chi-square (χ^2^), normed chi-square (χ^2^/*df*), comparative fit index (CFI), Tucker Lewis fit index (TLI), root mean square error of approximation (RMSEA), and standardized root mean square residual (SRMR). Values for CFI and TLI greater than 0.90, and RMSEA and SRMR lower than 0.08, are considered evidence of acceptable fit (Browne and Cudeck, 1993). Values for CFI and TLI close to 0.95, and RMSEA and SRMR lower than 0.05, are evidence of good fit (Hu and Bentler, 1999). Moreover, a χ^2^/*df* value less than 5 indicates an acceptable model fit (Schumacker and Lomax, 2004). Akaike’s Information Criterion (AIC) values were included as a measure for comparing the fit of alternative models. Improvements in model fits are reflected in higher values of CFI and TLI, and lower values of AIC, χ^2^, χ^2^/*df*, RMSEA, and SRMR.

After having ascertained the factorial validity of the measures, we performed path analysis to test whether PBS states mediate the link between students’ mastery and performance climate perception and motivation. To this purpose, we computed a self-determination index (SDI) score using the mean scores of the subscales of the SIMS, namely, IM, IR, ER, and AM. As indicated by several authors (e.g., Prusak et al., 2004; Ward et al., 2008; Johnson et al., 2011), we used the following formula: [SDI = +2(IM) +1(IR) -1(ER) -2(AM)]. Interpreting the SDI is straightforward: higher scores equate to higher levels of self-determined motivation. Likewise, we derived an index of PBS states subtracting scores of unpleasant/dysfunctional PBS states from scores of pleasant/functional PBS states. As can be observed in **Table 1**, mean scores of students’ pleasant/functional PBS states were substantially larger than scores of unpleasant/dysfunctional states. Thus, the higher the PBS index score, the higher the level of pleasant/functional PBS states experienced during the lessons.

**Table 1:** Descriptive statistics, correlations coefficients, alpha coefficients, and composite reliability values from Study 1.

Measure	*M*	*SD*	1	2	3	4	5	6	7	8
(1) Mastery climate	4.07	0.49	(0.73, 0.74)							
(2) Performance climate	2.03	0.68	-0.30^∗∗^	(0.79, 0.79)						
(3) Pleasant/functional PBS states	2.46	0.70	0.45^∗∗^	-0.15^∗^	(0.86, 0.82)					
(4) Unpleasant/dysfunctional PBS states	0.48	0.49	-0.34^∗∗^	0.39^∗∗^	-0.43^∗∗^	(0.79, 0.74)				
(5) Intrinsic motivation	5.05	1.03	0.59^∗∗^	-0.21^∗∗^	0.65^∗∗^	-0.46^∗∗^	(0.76, 0.76)			
(6) Identified regulation	5.19	1.02	0.38^∗∗^	-0.19^∗^	0.49^∗∗^	-0.29^∗∗^	0.67^∗∗^	(0.71, 0.73)		
(7) External regulation	2.21	1.22	-0.31^∗∗^	0.38^∗∗^	-0.23^∗∗^	0.51^∗∗^	-0.43^∗∗^	-0.32^∗∗^	(0.82, 0.82)	
(8) Amotivation	1.79	0.99	-0.29^∗∗^	0.47^∗∗^	-0.14	0.57^∗∗^	-0.32^∗∗^	-0.32^∗∗^	0.64^∗∗^	(0.83, 0.84)

*Alpha coefficients and composite reliability values are in brackets on the diagonal. ^∗^*p* < 0.05, ^∗∗^*p* < 0.01*.

Two simple mediation analyses were then conducted to examine in more detail whether the effects of perceived mastery climate on IM and IR were mediated by pleasant/functional PBS states. Similarly, two mediation analyses were performed to assess the effects of perceived performance climate on ER and AM through unpleasant/dysfunctional PBS states. We used the Hayes’ (2013) PROCESS computational tool for SPSS, which enables estimation of κ^2^ as a standardized index of effect size of simple effects that is insensitive to sample size, as well as bootstrap CIs for the indirect effects based on 5000 resamples (Preacher and Kelley, 2011).

### Results

In the data screening procedure, two multivariate outliers were identified using Mahalanobis’ distance criterion (*p* < 0.001 for the χ^2^ value), and subsequently removed. Thus, the final sample was comprised of 182 participants.

#### Descriptive Statistics of the Measures

Descriptive statistics, scale reliabilities, and correlation coefficients are reported in **Table 1**. Scores of perceived mastery climate, pleasant/functional PBS states, and IM/IR were larger than scores of perceived performance climate, unpleasant/dysfunctional PBS states, and ER/AM respectively.

It is interesting to note that mastery climate correlated positively with pleasant/functional PBS states, IM, and IR, while performance climate related positively to unpleasant/dysfunctional PBS states, ER, and AM. Moreover, positive relationships were found between IM and IR, and between ER and AM. All other interrelations among variables were negative and lower in magnitude. This pattern of correlations support the construct validity of the self-determination continuum wherein IM is associated with IR, and ER is related to AM appraisal (Ryan and Connell, 1989).

#### Factorial Validity of the Measures

Confirmatory factor analysis results for the TIMCPEQ, PBS states, and SIMS are reported in **Table 2**. CFA yielded acceptable fit indexes for the hypothesized factor structure of the instruments. Yet, examination of the modification indices for each measure suggested correlating two errors on each factor. This change led to substantial improvements of the model fit, thereby providing evidence for the factorial validity of the measures.

**Table 2:** Confirmatory factor analysis fit indices of the TIMCPEQ, the PBS states, and the SIMS models from Study 1.

Instrument	Model	*χ*^2^(*df*)	*χ*^2^/*df*	CFI	TLI	RMSEA (90% CI)	SRMR	AIC
TIMCPEQ	2-factors	82.124 (53)	1.550	0.936	0.920	0.055 (0.030–0.077)	0.054	5099.156
	2-factors and 2 correlated errors in each factor	65.587 (51)	1.286	0.968	0.958	0.040 (0.000–0.065)	0.050	5083.906
PBS states	2-factors	294.272 (76)	3.872	0.921	0.901	0.093 (0.077–0.110)	0.099	5546.774
	2-factors and 2 correlated errors in each factor	218.944 (74)	2.959	0.957	0.930	0.061 (0.038–0.082)	0.070	5345.587
SIMS	4-factors	236.625 (98)	2.415	0.916	0.902	0.083 (0.068–0.098)	0.085	8904.648
	4-factors and 2 correlated errors in each factor	212.672 (94)	2.262	0.932	0.926	0.034 (0.028–0.052)	0.054	8849.238

*χ^2^(*df*), chi-square (degree of freedom); CFI, comparative fit index; TLI, Tucker Lewis fit index; RMSEA, root mean square error of approximation; SRMR, standardized root mean square residual; AIC, Akaike’s Information Criterion*.

#### Path Analysis

**FIGURE 1:**
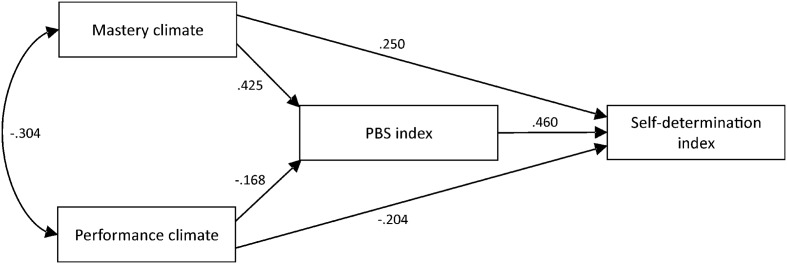
Standardized parameter values of the path model from Study 1. The path between performance climate and the PBS index is significant at *p* = 0.013. All other paths are significant at *p* < 0.001.

We conducted path analysis to test the model depicted in **Figure 1**. All standardized paths were significant at *p* < 0.001, with the exception of the path between performance climate and the PBS index that was significant at *p* = 0.013. Four simple mediation analyses were then conducted to examine in more detail whether the effects of perceived mastery and performance climates on motivation factors were mediated by PBS states. Findings showed that pleasant/functional PBS states partially mediated the effect of mastery climate on intrinsic motivation, κ^2^ = 0.242 (95% CI = 0.164–0.329), and IR, κ^2^ = 0.174 (95% CI = 0.105–0.255). Moreover, unpleasant/dyfunctional PBS states were found to partially mediate the effects of performance climate on ER, κ^2^ = 0.167 (95% CI = 0.078–0.267), and AM, κ^2^ = .186 (95% CI = 0.079–0.310). Preacher and Kelley (2011) contend that κ^2^ can be interpreted with CIs in terms of Cohen’s (1988) effect size indications for squared correlation coefficients. Values of 0.01, 0.09, and 0.25 represent small, medium, and large effect sizes, respectively. Thus, the observed mediation effects were medium-to-large given that CIs were > 0.09 and included 0.25.

### Discussion

Confirmatory factor analysis results supported the factorial validity of the measures. Moreover, results provided evidence for positive relationships among mastery climate, pleasant/functional PBS states, IM, and IR. Positive relationships were also observed among performance climate, unpleasant/dysfunctional PBS states, ER, and AM. Aligned with previous study findings (Bortoli et al., 2014), our analysis revealed that pleasant/functional PBS states partially mediated the path of mastery climate on IM and IR. Likewise, unpleasant/dysfunctional PBS states were found to partially mediate the path of performance climate on ER and AM. Overall, these findings (i.e., validity of instruments and relationships among variables) supported the use of the measures for the purposes of our experimental study (Study 2).

## Study 2

### Method

#### Participants

Using G^∗^Power software (Faul et al., 2007), *a priori* sample size calculation for a 2 (groups) by 3 (time points) between-within design, anticipating a medium effect size of 0.30, power level of 0.80 and *p* < 0.05, and non-sphericity correction of 0.70, indicated a total sample size of 26. Seventy female students aged 14–15 years (*M* = 14.50, *SD* = 0.50) from a high-school involved in Study 1 also took part in Study 2. The study was conducted with the permission of the headmaster. The students and their parents or guardians signed an informed consent in accordance with the Declaration of Helsinki.

#### Measures

We assessed the individual’s perceptions of the motivational climate, PBS states, and motivation, to determine the effectiveness of the intervention in creating a particular psychological climate and, as a consequence, in changing participants’ PBS states and motivation. To this purpose, we administered the same measures used in Study 1 (i.e., the TIMCPEQ, the list of PBS states, and the SIMS) across three time waves.

#### Design and Procedure

The design of the study included two experimental groups formed by two classes each. Five girls missed one or more lessons, and therefore were excluded from data analysis. The final sample (*N* = 65) included 30 girls in the first group (15 participants each class) and 35 girls in the second group (17 participants in a class and 18 in another class).

Following a comprehensive explanation of the study purposes, two female physical education teachers with over 10 years of teaching experience agreed to conduct 16 lessons. After being educated on the study’s purposes and methodology during a teacher-training course in physical education, they volunteered to take part in the study. The two teachers were knowledgeable about motor learning, skill development, and teaching styles (e.g., Mosston and Ashworth, 2008; Schmidt and Lee, 2014), but they did not have prior specific knowledge of or experience with the TARGET approach. The whole procedure included meetings with the teachers, three waves of data collection, and two intervention phases.

##### Peer-debriefing sessions

We organized three 2-h meetings with teachers before data collection and intervention, and four 1-h meetings during each of the intervention phases. Each meeting took place at the end of the week, after the two customary weekly lessons, for eight meetings in total. The purpose was to share with the teachers the rationale and the principles of the intervention and to design lesson plans. Theoretical and applied scholar writings on motivational climate, goal orientation, and IM were also presented. The methodological issues contained in these materials was thoroughly discussed with the teachers, especially the practical guidelines from the TARGET model (Epstein, 1989). The notions of task, authority, reward, grouping, evaluation, and timing were emphasized during each meeting. We also explained to the teachers that they had to adhere to the logic of a mastery climate or a performance climate, consistent with the pre-established phases of the intervention.

To promote and ensure compliance to the protocol, teachers were given evaluation logs on which to record for each lesson their adherence to the TARGET behaviors. For mastery or performance climates, the contents to be assessed included: (a) individualization of learning according to the students’ skill level and time needed to learn (mastery climate) vs. task progression based on predetermined teacher’s schedule (performance climate); (b) students’ involvement in decision-making vs. teacher making all the decisions; (c) private recognition of improvement and effort vs. public recognition of ability and performance in comparison with others; (d) formation of mixed ability and cooperative groups vs. differentiation of groups based on a similar ability level; (e) self-referenced and private evaluation vs. normative and public evaluation; and (f) flexible time for task completion vs. inflexible time for task completion. Personal evaluations were then examined and discussed during the weekly meetings. During the intervention, we videotaped a task- and an ego-involving lesson from each teacher and then provided feedback to them.

##### Data collection

The three assessment waves took place 2 days prior to or after the intervention. Specifically, one assessment occurred before the first intervention (T1; initial test), another in-between the first and the second intervention (T2; intermediate test), and the last at the end of the intervention (T3; final test). Assessments were conducted following the procedure outlined for Study 1.

##### Intervention

We adopted a quasi-experimental design given that it was impossible, in a real-world school environment, to have a random assignment of participants to the groups. Thus, intact classes formed the experimental groups. The study included two intervention phases of eight lessons each during 4 months, implemented after 2 months from the start of the academic year. The teachers were responsible for two classes: a class was initially involved in a mastery climate and later in a performance climate, while the other class was initially involved in a performance climate and later in a mastery climate. The two classes adopting a mastery climate formed the “mastery-performance group,” whereas the two classes adopting a performance climate formed the “performance-mastery group.” The mastery-performance and performance-mastery groups initiated the experimental activities simultaneously.

Along with the teachers, we prepared lesson plans to be applied in the same way for all classes. The main part of each lesson was preceded and followed by 5–10 min of warm-up and cool-down exercises. In the main part of a lesson, both groups were involved in learning and performing several basic gymnastics tasks often used in physical education as part of the academic curriculum. Fundamental gymnastic exercises for beginners included floor acrobatics, vaulting, forward and backward walks on the balance beam, and mini-trampoline jumps. Examples of floor acrobatics are forward roll, backward roll, assisted handstand, handstand and forward roll, handstand and backward roll, dive forward roll, and cartwheel. Vaulting included side, squat, and straddle vaults. Mini-trampoline exercises comprised straight, straddle, and tuck jumps. All activities were conducted under the supervision or direct assistance of the teacher. The main part of the lesson lasted about 30 min and contained at least three activities.

In planning activities, we adopted most of the indications deriving from the seminal TARGET model (Epstein, 1989), as well as other studies based on the TARGET model (e.g., Papaioannou and Goudas, 1999; Digelidis et al., 2003; Barkoukis et al., 2008; Morgan and Kingston, 2008). The lessons in the two groups were identical regarding the kind of activities taught and the amount of exercise. However, the two groups differed in the TARGET structures of the lessons. In the task-involving condition, tasks were designed to provide various levels of difficulty (e.g., progressing from forward roll to backward roll, or from handstand and forward roll to handstand and backward roll), and to be individualized according to one’s skill level and time needed to learn and improve. Students were encouraged to set specific and short-term goals, evaluate themselves on their own goals (i.e., self-referenced goals), and keep personal records to monitor personal improvement based on self-referenced criteria. They were also encouraged to participate in decision-making during the lessons (e.g., choosing the tasks, allocating time to the task, establishing intensity and frequency of engagement in an activity), work in pairs or form small groups of similar or mixed-ability in order to develop social interplay. The teacher provided private recognition to students for their personal effort, self-improvement, and achievement of personal goals.

In the ego-involving condition, students did not have the possibility to engage in individualized activities based on personal skill level, and progressed from one task to another following the teacher’s schedule and goals. Therefore, pupils were not encouraged to set goals, evaluate themselves, and participate in decision-making. Students were rewarded in public only when they were able to attain better achievements in comparison with their peers, whereas personal improvements, effort and participation remained substantially unnoticed.

After eight lessons and the intermediate data collection, the mastery-performance group participated in the ego-involving condition, while the performance-mastery group participated in the task-involving condition for additional eight lessons.

#### Data Analysis

Initially, data were screened for missing cases, outliers, skewness, kurtosis, linearity, and homoscedasticity (Tabachnick and Fidell, 2013). To examine the intervention effect, a 2 × 3 (group × test) repeated-measures analysis of variance (RM-ANOVA) was performed on the scores of mastery climate and performance climate, as well as on the PBS index and the SDI. The sources of significant effects on the PBS index and the SDI were then identified through *post hoc* comparisons using least significant difference (LSD) tests. Beyond the global indexes of PBS states and motivation, we were also interested in investigating possible differences between the two groups on the subscale scores of the PBS states and SIMS after the first phase of intervention. To this purpose, we conducted a multivariate analysis of variance (MANOVA) at T2. At this stage, differences between the two groups were expected to emerge.

Stepwise regression analysis was also conducted on the T2 data to determine which of the discrete-pleasant or unpleasant PBS states predicted individual’s motivation. Specifically, using the data gathered on the mastery-performance group, IM and IR were entered separately in the analysis as dependent variables, while pleasant/functional PBS items (discrete PBS states) were included as predictors. Analysis was conducted based on the positive relationships among mastery climate, IM, IR, and pleasant/functional PBS states, which were expected to emerge clearly in the mastery-performance group after the first phase of the intervention. With the data collected on the performance-mastery group, we ran regression analysis for ER and AM as dependent variables, and unpleasant/dysfunctional PBS items as predictors (according to the positive relationships expected among these variables).

### Results

**Table 3** contains mean variable scores (and *SD*) across the three phases of the assessment. RM-ANOVA results are presented in **Table 4**. As can be seen, all group × test interaction findings were significant. *Post hoc* comparisons yielded significant differences. Mean scores of the mastery-performance group on both perceived mastery and performance climate did not differ significantly from T1 to T2. Yet, mastery climate scores decreased while performance climate scores increased from T1 and T2 to T3 (*p* = 0.003 and *p* < 0.001, respectively). In contrast, mean scores of the performance-mastery group on mastery climate decreased from T1 to T2 and increased from T2 to T3 (*p* < 0.001 and *p* = 0.013, respectively), while mean scores on performance climate increased from T1 to T2 (*p* = 0.011). The changes in the perceived motivational climate were reflected in the SDI and the PBS index in the participants of the performance-mastery group who decreased their SDI scores from T1 to T2 (*p* < 0.001), and from T1 to T3 (*p* = 0.030). PBS index scores were also lower at T2 compared to T1 (*p* = 0.030) for this group. Of note, compared to the mastery-performance group, participants in the performance-mastery group at T2 reported lower scores on mastery climate (*p* = 0.002), SDI index (*p* = 0.004), and PBS index (*p* = 0.027), and higher scores on performance climate (*p* = 0.010). These between groups differences, due to the changes in the variable scores from T1 to T2 in the performance-mastery group (see **Table 3**), were not observed at T3.

**Table 3:** Mean and standard deviation of variable scores across the three assessment phases from Study 2.

Dependent variable	Mastery-performance group			Performance-mastery group		
	Initial test *M (SD)*	Intermediate test *M (SD)*	Final test *M (SD)*	Initial test *M (SD)*	Intermediate test *M (SD)*	Final test *M (SD)*
Mastery climate	4.15 (0.60)	4.14 (0.40)	3.71 (0.61)^d^	4.13 (0.37)	3.63 (0.71)^a^	3.99 (0.50)^c^
Performance climate	1.83 (0.68)	1.77 (0.64)	2.41 (1.02)^d^	1.86 (0.53)	2.27 (0.98)^a^	1.99 (0.64)
Pleasant/functional PBS states	2.45 (0.77)	2.60 (0.57)	2.58 (0.70)	2.42 (0.63)	2.22 (0.60)	2.40 (0.73)
Unpleasant/dysfunctional PBS states	0.42 (0.37)	0.36 (0.30)	0.42 (0.41)	0.41 (0.34)	0.51 (0.42)	0.49 (0.51)
PBS index	2.03 (0.93)	2.23 (0.79)	2.16 (0.98)	2.02 (0.88)	1.71 (0.93)^a^	1.92 (1.06)
Intrinsic motivation	5.17 (1.06)	5.42 (0.89)	4.98 (0.93)	5.17 (0.79)	4.94 (1.15)	5.01 (1.11)
Identified regulation	5.28 (1.01)	5.23 (1.17)	4.90 (1.12)	5.39 (0.89)	4.69 (1.31)	4.96 (1.00)
External regulation	1.95 (0.94)	2.19 (1.12)	2.11 (0.95)	1.97 (1.08)	2.37 (1.18)	2.21 (1.16)
Amotivation	1.53 (0.62)	1.35 (0.49)	1.51 (0.69)	1.62 (0.60)	2.04 (1.31)	1.88 (0.84)
Self-determination index	10.60 (4.06)	11.17 (3.64)	9.74 (3.79)	10.51 (3.44)	8.12 (5.43)^a^	9.01 (4.25)^b^

*Significant differences (*p* < .05) between tests:*^a^*initial vs. intermediate*, ^b^*initial vs. final*, ^c^*intermediate vs. final*, ^d^*both initial and intermediate vs. final*.

**Table 4:** 2 × 3 (Group × Test) repeated-measures analysis of variance results from Study 2.

Dependent variable	Effect	*F* (*df*)	*p*	$\eta_{p}^{2}$	Power
Mastery climate	Group	0.696 (1, 63)	0.407	0.011	0.130
	Test	7.847 (2, 126)	<0.001	0.111	0.948
	Group × Test	12.535 (2, 126)	<0.001	0.166	0.996
Performance climate	Group	0.070 (1, 63)	0.793	0.001	0.058
	Test	4.542 (2, 126)	0.012	0.067	0.764
	Group × Test	7.757 (2, 126)	<0.001	0.110	0.946
Psychobiosocial states index	Group	1.653 (1, 63)	0.203	0.026	0.244
	Test	0.226 (2, 126)	0.798	0.004	0.085
	Group × Test	3.458 (2, 126)	0.035	0.051	0.590
Self-determination index	Group	2.219 (1, 63)	0.141	0.034	0.311
	Test	3.87 (2, 126)	0.037	0.049	0.587
	Group × Test	4.817 (2, 126)	0.010	0.071	0.790

MANOVA on the subscale scores of PBS states and SIMS at T2 yielded significant results, Wilks’ λ = 0.802, *F*(6,58) = 2.380, *p* = 0.040, $\eta_{p}^{2}$ = 0.198, power = 0.772. Between-groups follow-up showed that the performance-mastery group scored lower on pleasant/functional PBS states, *F*(1,63) = 6.513, *p* = 0.013, $\eta_{p}^{2}$ = 0.094, power = 0.710, and IM, *F*(1,63) = 4.127, *p* = 0.046, $\eta_{p}^{2}$ = 0.063, power = 0.540, and scored higher on AM, *F*(1,63) = 7.486, *p* = 0.008, $\eta_{p}^{2}$ = 0.106, power = 0.769.

Regression analysis results are summarized in **Table 5**. The adjectives “socializing, collaborative,” and “happy, joyful, cheerful,” representing communicative and emotional PBS states, were shown to predict IM and IR in the mastery-performance group. In contrast, the adjectives “unmotivated, disengaged” and “inactive, sluggish, passive,” representing motivational and cognitive PBS states, predicted ER and AM in the performance-mastery group.

**Table 5:** Psychobiosocial (PBS) states as predictors of intrinsic motivation, identified regulation, external regulation, and amotivation.

Group, motivation, PBS states	*β*	*R*^2^	*R*^2^ change	*F* change	*F* sig. change
**Mastery-performance group**					
Intrinsic motivation					
Socializing, collaborative (communication)	0.607	0.457	0.457	23.580	0.000
Happy, joyful, cheerful (emotion)	0.319	0.554	0.097	5.846	0.023
Identified regulation					
Socializing, collaborative (communication)	0.590	0.349	0.349	14.985	0.001
**Performance-mastery group**					
External regulation					
Unmotivated, disengaged (motivation)	0.438	0.212	0.212	8.900	0.005
Inactive, sluggish, passive (cognition)	0.320	0.314	0.102	4.748	0.037
Amotivation					
Unmotivated, disengaged (motivation)	0.463	0.214	0.214	9.011	0.005

### Discussion

Results on perceived motivational climate indicate that the program was effective in creating a particular psychological atmosphere (i.e., task- or ego-involving) after the first and/or second intervention phases. The changes were also reflected in PBS states and self-determined motivation in the performance-mastery group. Results and inspection of the mean scores across T1, T2, and T3 (see PBS index and SDI scores in **Table 3**) indicate that participants in the performance-mastery group experienced lower levels of pleasant/functional PBS state and self-determined motivation from T1 to T2. This group also reported SDI scores at T3 significantly lower than T1, thereby suggesting enduring effects of an ego-involving experience.

Altogether, findings support the effectiveness of the intervention in manipulating the perceived motivational climate. The related changes on PBS states and motivation were more pronounced in the performance-mastery group than the other group.

## General Discussion

The main aim of this study was to investigate the effect of mastery and performance climate manipulations on students’ climate perception, PBS states, and self-determined motivation using a counterbalanced design in a real-world setting. In Study 1, CFA findings provided evidence for the factorial validity of the measures then used in Study 2. Moreover, pleasant/functional PBS states were shown to partially mediate the effect of mastery climate on IM and IR, while unpleasant/dyfunctional PBS states were found to partially mediate the effect of performance climate on ER and AM. These results are in line with Bortoli et al. (2014) results suggesting that a mastery atmosphere in physical education improves students’ self-determined motivation through the mediation of pleasant/functional states, whereas a performance atmosphere depresses self-determined motivation through the mediation of unpleasant/dysfunctional states.

The intervention implemented in Study 2 was grounded in the six dimensions of the TARGET model (task, authority, recognition, grouping, evaluation, and timing). The model was applied to create a mastery climate (with emphasis placed on effort, personal improvement, self-referenced goals, and cooperation), or a performance climate (with a focus on outcome, appreciation mainly of the best students, and interpersonal competition). A group took part in eight task-involving lessons, and then, in a second phase, in eight ego-involving lessons (mastery-performance group). The other group took part in ego-involving lessons and then in task-involving lessons (performance-mastery group). This counterbalanced design allowed us to investigate the impact of different motivational climates on students’ emotional states and motivation.

At the initial test, students in both groups reported variable scores very similar in magnitude (see **Table 3**), higher in perceived mastery climate, pleasant/functional PBS states, and IM compared to perceived performance climate, unpleasant/dysfunctional PBS states, and external motivation. Mastery climate correlated positively with pleasant/functional PBS states, IM, and IR, while performance climate was positively related to unpleasant/dysfunctional PBS states, ER, and AM. Correlation results corroborate findings of previous studies in physical education research grounded in achievement goal theory and self-determination theory (Standage et al., 2003; Liukkonen et al., 2010), and support the view that a mastery climate is functional in the school context. This is in accordance with the educational goals that characterize physical education programs in Italy and the pedagogical aspects that teachers often emphasize (Italian Ministry of Education, University, and Research, 2009).

Regarding motivational climate manipulation, Hypothesis 1 was partially confirmed. As expected, a performance atmosphere influenced students’ climate perception. After the first intervention phase, at the intermediate test (T2) the performance-mastery group reported lower scores on perceived mastery climate (and higher scores on perceived performance climate) compared to the initial scores (T1). However, students in the mastery-performance group did not change their perceptions of mastery climate from T1 to T2. This lack of significant changes between T1 and T2 can be likely attributed to the mastery atmosphere of the experimental condition similar to the motivational climate commonly found in regular courses. Indeed, teacher education programs in Italy place emphasis on motor skill development, as well as on knowledge and behaviors associated with out-of-school engagement in sport and physical activity (Italian Ministry of Education, University, and Research, 2009). Teachers usually pursue these educational goals in a supportive learning environment more related to individual progresses and cooperation within peers than to performance outcomes and competition. Despite the lack of changes from T1 to T2, the mastery-performance group at T2 reported higher scores on mastery climate perceptions and lower scores on performance climate perceptions than the performance-mastery group. At the end of the second phase of the intervention (T3), students in both groups significantly changed their perception of mastery climate as predicted. This pattern of results indicates that both interventions were effective in influencing students’ climate perception.

The motivational climate manipulation determined changes on PBS states and self-determined motivation, particularly in the performance-mastery group. Participants in this group decreased their PBS index and SDI scores from T1 to T2 (Hypothesis 2). A detailed between-subjects analysis at T2 showed lower levels of pleasant/functional PBS states and IM, and higher levels of AM in the performance-mastery group. Contrary to our expectations, we did not find significant changes on PBS states and self-determined motivation at T3 deriving from changes in the motivational atmosphere (Hypothesis 3). It is important to note that participants in the performance-mastery group did not improve their level of self-determination from T2 to T3, notwithstanding a task-involving intervention (i.e., the SDI scores at T3 remained significantly lower than T1). These results suggest a carryover detrimental effect of an ego-involving experience, likely because of enduring feelings of disengagement or frustration arising from a performance climate.

From an applied perspective, our findings suggest that teachers should refrain from adopting a performance climate in their classes from the very start, in order to prevent long lasting harmful effects on students’ self-determined motivation. Results support the positive relationship between perceived mastery climate and IM, and provide evidence that teachers can influence students’ self-determined motivation by creating a mastery motivational climate (e.g., Cury et al., 1996; Jaakkola and Liukkonen, 2006; Sproule et al., 2007). Deci and Ryan (2000) recognized the general convergence of evidence from achievement goal theory and self-determination theory regarding the optimal design of learning environments. Both theories posit that IM is nurtured in environments that promote choice and desire to learn rather than social comparisons, normatively based goals, reward provided contingent on performance. Sproule et al. (2007) contended that teachers are able to enhance students’ self-determined motivation by creating a mastery environment. In this pedagogical context, students tend to increase their perception that their actions and decisions in physical education are under their own control. A mastery climate can foster students’ need to be self-determined, and enhance their perception of engaging in an activity volitionally, which in turn increases self-determined motivation.

A specific motivational climate has also emotional consequences. Our findings concur with those of previous studies in physical education grounded in the achievement goal theory and implementing different motivational climate interventions. With respect to PBS states, Bortoli et al. (2015) observed lower scores in pleasant/functional PBS states and higher scores in unpleasant/dysfunctional PBS states in a performance group compared to a mastery group. More generally, the meta-analytic review conducted by Braithwaite et al. (2011) revealed that emotional adaptive outcomes (such as enjoyment, commitment, and confidence) are more likely to occur in mastery climate conditions, whereas maladaptive outcomes (such as anxiety and boredom) are more commonly reported in performance climate conditions. Indeed, emphasizing social comparison and doing better than others, rather than individual efforts, attainments, and mastery of skills, can create feelings of worry and apprehension. Demonstrating low competence to others and public situations of failure can result in negative emotional experiences.

Descriptive and inferential analysis of changes to PBS states provide further insight into the intervention effects. At the end of the first phase of the treatment, regression analyses showed specific pleasant/functional states to predict IM and IR in the mastery-performance group. The adjectives “socializing, collaborative,” and “happy, joyful, cheerful,” representing communicative and emotional PBS states, highlight the value of relatedness and emotion on self-determined motivation. On the other hand, the adjectives “unmotivated, disengaged” and “inactive, sluggish, passive,” indexing motivational and cognitive PBS states, predicted ER and AM in the performance-mastery group.

## Conclusion

Taken together, our findings showed that the manipulation of TARGET dimensions was effective in creating a particular motivational climate and in influencing emotional and motivational states. The practical implication is that physical education teachers need to be aware of the detrimental effects of a performance climate in their classes, and therefore should carefully consider the way they structure the lessons. It is worth mentioning that Morgan and Kingston (2008) have developed a mastery intervention program for teacher education based on the TARGET model. This and other programs can be used and developed to help teachers learn how to design healthier motivational climates for their students of various backgrounds and personal characteristics.

In conclusion, our findings support the relationship between pupils’ perceived motivational climate, emotional states, and self-determined motivation. A limitation of this investigation (in particular with Study 2) is the quasi-experimental design. However, it should be considered that our purpose was to examine the effects of a program in a real-world setting where it is not feasible to randomly assign participants to different experimental groups. Other limitations involve the lack of a control group, the lack of information on long-term intervention results and generalizability of findings, and the inclusion of female participants only. The influence of personal variables should also be examined, including actual and perceived competence, previous sporting experience, and current involvement in sport. Thus, future research should include a control group, examine long-term outcomes of intervention programs on both female and male participants, investigate generalizability of effects to out-of-school contexts, and consider the effect of personal factors. Several studies have shown that perceived mastery climate, pleasant emotional states, and IM in physical education lessons can boost individuals’ intention to be physically active and to practice sport and exercise in their leisure time (Escartí and Gutiérrez, 2001; Sproule et al., 2007). Consequently, teachers’ instructional style in physical education aligned with the TARGET model tenets is expected to enhance positive emotions and IM during the lessons, facilitate positive attitudes toward outside school physical activity, and promote health habits in youth.

## Ethics Statement

Participants in the study and their parents or guardians signed an informed consent in accordance with the Declaration of Helsinki. Ethical approval for the study was obtained from the ethics committee for biomedical research of the “G. d’Annunzio” University of Chieti-Pescara, Italy, with anonymity and confidentiality being assured for all the participants.

## Author Contributions

All authors listed have made a substantial, direct and intellectual contribution to the work, and approved it for publication.

## Conflict of Interest Statement

The authors declare that the research was conducted in the absence of any commercial or financial relationships that could be construed as a potential conflict of interest.
